# Short-Term Outcomes of First 100 Laparoscopic Colorectal Surgeries at a Newly Developed Surgical Setup at Peshawar

**DOI:** 10.7759/cureus.53588

**Published:** 2024-02-04

**Authors:** Muhammad F Shah, Irfan Ul Islam Nasir, Riaz Ahmad, Sajjad Ahmad, Aalia Amjad, Khush Bakht Zaineb, Romana Rehman

**Affiliations:** 1 Surgical Oncology, Shaukat Khanum Memorial Cancer Hospital and Research Centre, Peshawar, PAK; 2 Colorectal Surgery, Shaukat Khanum Memorial Cancer Hospital and Research Centre, Peshawar, PAK; 3 Medicine, Shaukat Khanum Memorial Cancer Hospital and Research Centre, Peshawar, PAK

**Keywords:** tertiary care center, surgical setup, short-term outcomes, minimally invasive surgery, colorectal cancer, laparoscopy

## Abstract

Background: The incidence of colorectal cancer (CRC) has risen steadily, necessitating innovative strategies for diagnosis and treatment. Minimally invasive surgery, exemplified by laparoscopic techniques, has emerged as a transformative approach in colorectal surgical practices. Laparoscopy offers advantages such as improved aesthetic outcomes, reduced post-operative pain, early patient mobilization, and shorter hospital stays.

Objective: This study aims to present the short-term surgical outcomes of the first 100 elective laparoscopic CRC resections performed at a newly established tertiary care cancer center in Peshawar, Pakistan.

Materials and methods: Data were prospectively collected for CRC resections performed between April 2021 and February 2022. The study included patients above 18 years of age with biopsy-proven CRC. Surgical procedures were performed by two dedicated colorectal surgeons trained in minimally invasive surgery. Patient demographics, pre-operative factors, intraoperative parameters, and post-operative outcomes were systematically recorded and analyzed.

Results: Among the 100 cases included in the study, laparoscopic colorectal surgeries were successfully performed without any conversions to open surgery. The mean age of the study population was 52.5 years, with a male-to-female ratio of 2:1. The majority of cases were colon (48%) and anorectal cancers (52%). The mean lymph node yield was 18.29 (range 6-49). Only one patient required a re-look laparoscopy for a pelvic hematoma, and overall mortality was reported at 1%.

Conclusion: Laparoscopic colorectal surgery is a safe and effective treatment option for elective colorectal operations with minimal post-operative complications and favorable short-term outcomes.

## Introduction

Colorectal cancer (CRC) is a formidable global health challenge, ranking as the second most common cancer in Pakistan and the third leading cause of cancer-related mortality worldwide [1,2]. This malignancy, arising from the colon or rectum, demands innovative strategies for diagnosis and treatment to enhance patient outcomes. The arena of CRC management has witnessed a transformative evolution since the introduction of minimally invasive surgery (MIS) in 1985 [3].

Accurate diagnosis is paramount in navigating the complexities of CRC. Screening methods, including colonoscopies and advanced imaging techniques, play a pivotal role in early detection [4]. The evolving diagnostic landscape incorporates molecular and genetic profiling, facilitating personalized treatment strategies. As CRC often presents asymptomatically in its early stages, a comprehensive diagnostic approach is crucial for timely intervention.

Laparoscopic surgery, a hallmark of MIS, has emerged as a convincing alternative to traditional open resections for CRC [5]. While studies affirm the non-inferiority of laparoscopy in long-term outcomes, its adoption faces challenges due to the demanding learning curve [6-8]. The effectiveness of laparoscopic techniques lies not only in their safety, validated through randomized control trials, but also in their potential to improve short-term outcomes, revolutionizing surgical practices [9-11].

Against this context, our study undertakes a detailed examination of the initial 100 elective CRC resections at a state-of-the-art cancer unit in Peshawar, Pakistan. This study explores the use of laparoscopic techniques in a region where CRC is a major healthcare challenge. By providing insights into surgical innovations, our research aims to evaluate the short-term outcomes of the first 100 colorectal surgeries at a newly developed tertiary care cancer center.

## Materials and methods

The data was collected prospectively for CRC resections performed between April 2021 and February 2022 at the newly established Surgical Oncology Department, Shaukat Khanum Memorial Cancer Hospital and Research Centre (SKMCH&RC) in Peshawar, Pakistan. Institutional Review Board (IRB) approval to conduct the study was obtained (reference # Ex-04-06-22-01). Prior to surgery, written informed consent was taken from all the patients. All the surgeries were performed by two dedicated colorectal surgeons who were trained in minimally invasive surgery.

Data collection

Patients of both genders above 18 years of age who had biopsy-proven CRC were included. According to our institutional policy, all cases were discussed in multidisciplinary team meetings (MDT) before the initiation of treatment. Patients less than 18 years old and those with secondary extension to the colon or rectum were excluded from the study. Furthermore, patients with stage IV tumors were excluded from the study based on CT-Scan findings.

Pre-operative factors like age, gender, body mass index (BMI), ASA scores, comorbidities, previous surgery, baseline CEA, number of tumors found on colonoscopy, clinical stage, and neoadjuvant chemotherapy in rectal cancers were included in the study. Intraoperative parameters like operative time, stoma formation, blood loss, and intraoperative complications were included. Post-operative outcomes like the length of stay, post-operative complications, assessment of circumferential resection margins in rectal cancers, and histological type and stage were included. Furthermore, mortality within 30 days and readmission within 30 days were recorded.

Surgical technique

**Figure 1:**
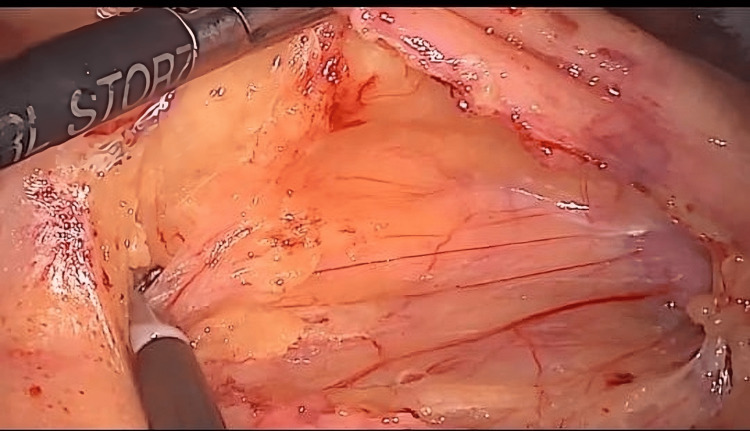
Creation of ileocolic window with medial to lateral dissection

The procedure was performed for right-sided tumors of the colon using four ports. The optical port (11 mm) was placed below the umbilicus and used for creating pneumoperitoneum by the Hasson technique. A laparoscopy was then performed, and the peritoneal cavity was inspected for any metastatic disease and for confirmation of the site of the tumor. One 12-mm working port was then placed under vision in the left iliac fossa followed by the placement of two 5-mm ports, one in the suprapubic region and the other in the left hypochondrium in the midclavicular line below the costal margin. The patient was placed in the Trendelenburg position and the table was tilted to the left. The small bowel was pushed medially, and a gauze was placed on it to clear the operating field. The dissection started after assessing the resectability of the tumor. The ileocolic window was created, and the ileocolic vessels were clipped and ligated, followed by medial to lateral dissection till the lateral abdominal wall (Figure 1).

**Figure 2:**
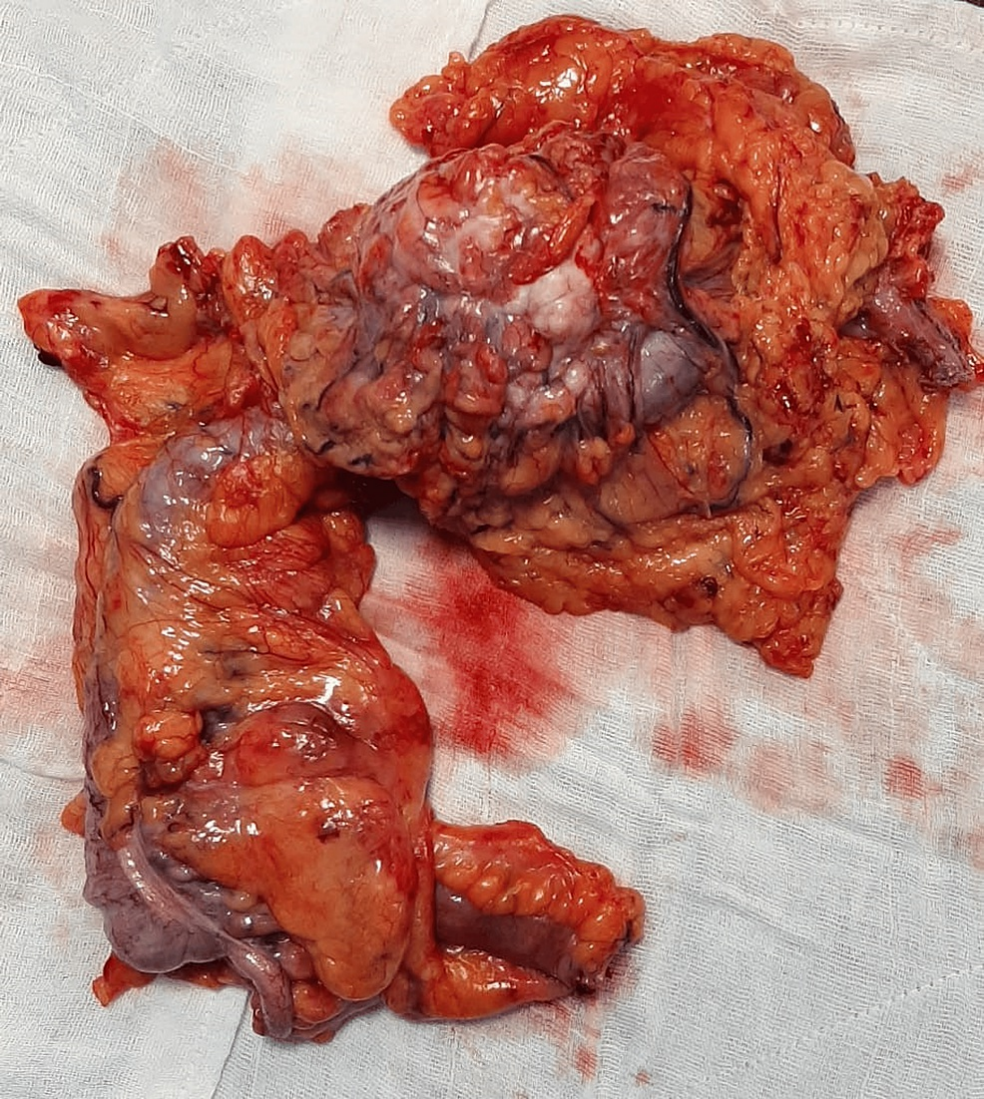
Proximal transverse colon tumor

Subileal dissection was performed with care taken to avoid injury to the ureter, gonadal vessels, and duodenum. The gastrocolic ligament was dissected, the lesser sac opened, and complete mobilization of the hepatic flexure was performed. The right branch of the middle colic artery was dissected, clipped, and transected. A small right subcostal transverse incision was made, the specimen was retrieved, and extracorporeal anastomosis was performed (Figure 2). Wounds were closed in reverse order following the closure of the port sites.

**Figure 3:**
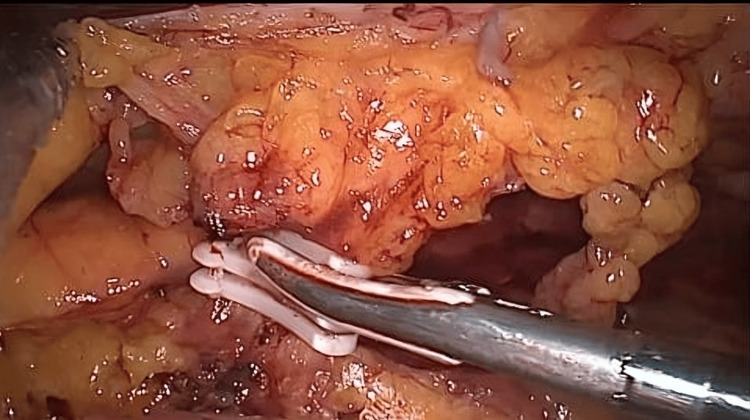
Clipping and transection of Inferior mesenteric artery

Surgeries for tumors on the left side of the colon and in the pelvis were conducted using five ports. An 11-mm optical port was placed above the umbilicus using an open technique, and pneumoperitoneum was established (maintaining 12-15 mm Hg pressure and 25-30 L of CO_2_ flow). The peritoneal cavity was assessed for any metastatic disease. A 12-mm working port was placed in the right iliac fossa, 3 cm medial and above the anterior superior iliac spine. Three 5-mm ports were placed under vision; one in each of the right and left flanks and one in the suprapubic region. The patient was positioned in the Trendelenburg position, and the table was tilted to the right. The dissection of the sigmoid mesocolon was initiated from the medial to the lateral side, followed by skeletonizing, clipping, and transection of the inferior mesenteric artery (Figure 3).

**Figure 4:**
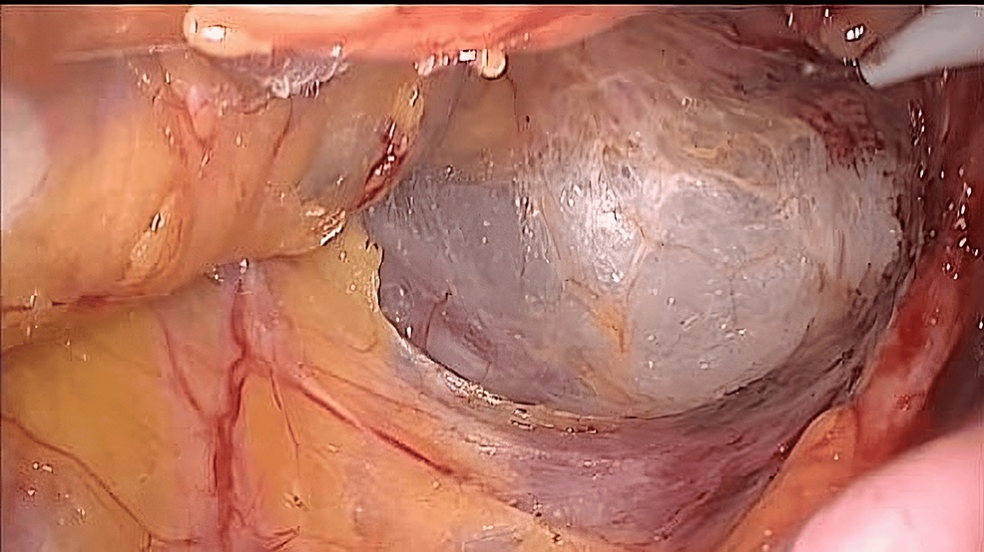
Posterior total mesorectal dissection

Medial to lateral dissection was continued until the lateral abdominal wall, with careful consideration of the ureter and gonadal vessels. Then, total mesorectal dissection was initiated circumferentially in the pelvis, starting posteriorly followed by lateral and anterior dissection down to the pelvic floor, while taking care to preserve the inferior hypogastric plexus, ureter, and major vessels (Figure 4).

The transection was performed at the proper place of the descending colon. The perineal part began after making an elliptical incision, dissecting the ischiorectal fats, and levator ani muscles. The specimen was retrieved by the somersault maneuver (this technique in APR involves perineal dissection of the specimen posteriorly and laterally while still attached anteriorly. Then, the specimen is delivered outside the body and the remaining attached anterior part of the specimen is dissected). Proper closure of the wound was done after washing it with normal saline. Dressing was performed. A permanent stoma was fashioned in the left iliac fossa on the already marked stoma site following the closure and dressing of the port sites.

Statistical analysis

Statistical analysis was performed on IBM SPSS Statistics for Windows, Version 26 (Released 2019; IBM Corp., Armonk, New York). For continuous variables, descriptive statistics were calculated and expressed as medians and ranges.

## Results

A total of 100 patients were enrolled in the study. Age ranged between 20 and 85 years, with the mean age being 52.5 ± 17.1 years. Of the patients, 67 (67%) were male and 33 (33%) were female, with a male-to-female ratio of 2:1. A total of 93 (93%) of the patients were classified as ASA-II, while ASA-III and ASA-IV were 6 (6%) and 1 (1%), respectively. Among the 100 cases, 72 (72%) belonged to Khyber Pakhtunkhwa (KPK), 21 (21%) from Afghanistan, 5 (5%) from the Federally Administered Tribal Areas (FATA), and 2 (2%) from Punjab (Table 1).

**Table 1:** Demographics of the patients ASA: American Society of Anesthesiologists, BMI: body mass index, KPK: Khyber Pakhtunkhwa, FATA: Federally Administered Tribal Areas, SD: standard deviation.

Demographics	Frequency, n (%)
Gender	
Male	67 (67%)
Female	33 (33)
ASA class	
ASA-I	0 (0%)
ASA-II	93 (93%)
ASA-III	6 (6%)
ASA-IV	1 (1%)
BMI status	
Below 18.5 kg/m^2^	8 (8%)
18.5-24.9 kg/m^2^	57 (57%)
25-29.9	26 (26%)
<30 kg/m^2^	9 (9%)
Region	
KPK	72 (72%)
Afghanistan	21 (21%)
FATA	5 (5%)
Punjab	2 (2%)
Mean age (mean ± SD)	52.5±17.1 years

The most common procedure performed was abdominoperineal excision (APR) with 23 patients. APR was done because of the involvement of the sphincter and its poor function. Right hemicolectomy was performed in 22 patients; all anastomoses were performed extracorporeally. Those with low anterior resection and ultra-low anterior resections were defunctioned by proximal covering ileostomy. Hartmann’s procedure was carried out in 7 patients, among which 3 patients had metastatic rectosigmoid cancer, and the rest of the 4 patients had thickened edematous mesentery with massively dilated colon not fit for anastomosis.

One patient had a relook laparoscopy and washouts because of a pelvic hematoma. Mortality occurred in one patient (1%) because of aspiration pneumonia during the 30-day postoperative period. A total of 94 (94%) patients had R0 resection while 6 (6%) had R1 resections who had involved circumferential resection margin even after neoadjuvant treatment for rectal cancers (Table 2).

**Table 2:** Procedures performed Rt: right, APR: abdominoperineal resection, R0: no microscopic involvement, R1: microscopic involvement.

Procedure	Frequency, n (%)
Pan proctocolectomy	3 (3%)
Total colectomy	5 (5%)
Rt hemicolectomy	22 (22%)
Hartman’s	7 (7%)
Sigmoid colectomy	17 (17%)
Ultra-low anterior resection	14 (14%)
APR	23 (23%)
Extra levator APR	9 (9%)
Resection type	
R0 resection	94(94%)
R1 resection	6 (6%)
Overall mortality	1(1%)

Most of the tumors at presentations were T3 77(77%), followed by T4 15(15%), T2 7(7%), T1 0(0%), and T0 1(1%). Similarly, N stage 0 was recorded in 10(10%) cases, followed by N1 58(58%) cases and N2 in 32(32%) cases, respectively (Table 3).

**Table 3:** Stage of tumor (T-stage and N-stage) T1: tumor involvement submucosa, T2: tumor invades muscularis propria, T3: tumor involves submucosa, T4: tumor involvement other adjacent organs/structures, N0: no nodal involvement, N1: metastasis in 1-3 lymph nodes, N2: metastasis in 4-7 lymph nodes, N3: metastasis in apical lymph nodes.

Stage	Frequency, n (%)
“T” stage	
T0	1 (1%)
T1	0 (0%)
T2	7 (7%)
T3	77 (77%)
T4	15 (15%)
“N” stage	
N0	10 (10%)
N1	58 (58%)
N2	32 (32%)

All 100 surgeries were successfully performed using laparoscopic techniques, and there was no conversion from laparoscopic to open surgery. The rectum was identified as the most common site of cancer in 52 (52%) of the cases, followed by the ascending and sigmoid colon, each with 21 (21%) cases. Of the sigmoid colon cases, 17 patients underwent sigmoid colectomy, while the remaining 4 patients, along with 2 patients with descending colon tumors, underwent pan-proctocolectomy and total colectomy due to the presence of synchronous tubulovillous adenoma with high-grade dysplasia (Table 4).

**Table 4:** Tumor location

Tumor location	Frequency, n(%)
Rectum	52 (52%)
Ascending colon	21 (21%)
Sigmoid	21 (21%)
Transverse colon	4 (4%)
Descending colon	2 (2%)

In only 1 (1%) case, aspiration pneumonia was observed. No anastomotic leak or other major complications were observed during the study period.

The mean length of stay was 6.3 ± 1.9 days (range 4-8 days). The mean intra-operative blood loss was 24.5 ± 3.2 ml (range 20-30 ml). Five patients required blood transfusions intraoperatively, accounting for 5% of the cases. The mean number of lymph nodes examined was 18.29 ± 7.8 (range 6-49). A total of 44 (44%) patients had received neoadjuvant chemotherapy. Overall, 98 (98%) patients were diagnosed with adenocarcinoma. The other histological types observed were adenosquamous carcinoma in 1 (1%) case and rhabdomyosarcoma in 1 (1%) case (Table 5).

**Table 5:** Intra-operative and post-operative characteristics

Characteristics	Mean ± SD
Length of stay	6.3±1.9 days
Intraoperative blood loss	24.5±3.2 ml
Lymph nodes, mean	18.29±7.8
	Frequency, n (%)
Blood transfusion	5 (5%)
Neo-adjuvant chemo	44 (44%)
Histological types	
Adenocarcinoma	98 (98%)
Adenosquamous carcinoma	1 (1%)
Rhabdomyosarcoma	1 (1%)

## Discussion

Despite the safe utilization of advanced laparoscopic surgery for diverse procedures across multiple medical centers, it has not reached the status of being considered the gold standard in colorectal surgery [12]. The benefits associated with minimally invasive surgery, such as an improved aesthetic outcome, less post-operative pain, early patient mobilization, shorter hospitalization, lower morbidity rates, and an early return to work, are widely acknowledged [13,14]. Consequently, it is increasingly recognized as the preferred treatment option owing to its widespread availability in surgical practices [15]. We have reviewed the outcomes of our first 100 colorectal cases to ensure the safety and quality of our newly established Department of Surgical Oncology.

The age of study patients was 20-85 years mean (±52.5), which is comparable to other studies [16,17]. The youngest patient we had presented with per rectal bleeding and altered bowel habits, on MRI sigmoid carcinoma predicted stage of T3 N1 MX. Various studies have been investigating the increasing incidence of CRC in the younger age group, particularly among young adults aged 20-49 years [18,19]. Between 1975 and 2014, there were 70,400 incident cases of CRC diagnosed [20]. Overall, the incidence rate increased from 9.9 per 100,000 in 1975-80 to 11.7 per 100,000 in 2010-14, with Yale medicine and colorectal surgery doctors also reporting an increase in young-onset CRC, noting the youngest CRC patient diagnosed in recent months was 18 [21].

In our study, male patients were predominant compared to females. Based on location, the rectum was the most common site followed by the ascending colon and sigmoid. In assessing intra-operative safety, our focus centered on blood loss and conversion rates. Notably, only 5% of patients required blood transfusion during surgery. A distinctive highlight of our research lies in the exclusive use of laparoscopic procedures, achieving a remarkable zero conversion rate and anastomotic leak rate.

The post-operative mortality rate, while minimal, was reported in only one patient. The average hospital stay for our study cohort was 6.3 days (ranging from 4 to 8 days). It is worth noting that slightly prolonged hospitalization was observed in one patient due to aspiration pneumonia. Our findings align with the study of Kim SJ et al., indicating a reduced hospital stay and accelerated recovery in laparoscopically treated patients [22].

Remarkably, our recorded data showcases a substantial lymph node yield, averaging 17.25 (ranging from 6 to 49). These compelling results underscore the safety and efficacy of our laparoscopic approach, contributing to favorable patient outcomes.

While laparoscopic colorectal surgery offers numerous advantages for patients, it poses technical challenges for surgeons and their teams. Despite the steep learning curve associated with laparoscopy, even relatively new surgical centers can establish laparoscopic units with minimal repercussions. We believe that surgical duration tends to decrease with increasing laparoscopic experience, a sentiment echoed in the literature where approximately 20-30 procedures are deemed necessary for a surgeon to be considered experienced.

Our findings indicate that a recently established laparoscopic setup can undertake colorectal procedures with minimal morbidity and mortality. The successful initiation of our laparoscopy unit and favorable outcomes in laparoscopic colorectal surgeries align with results from other centers in the literature.

It is important to acknowledge the limitations of our analysis, primarily stemming from the short-term data collection that fails to assess the long-term outcomes of patients. Results may vary based on surgical experiences and the physical condition of patients. Additionally, this study is confined to a single institution without a comparison group, which warrants consideration when interpreting the findings.

## Conclusions

In conclusion, our study on the short-term outcomes of the first 100 laparoscopic CRC resections at a newly established tertiary care cancer center in Peshawar demonstrates the safety and efficacy of this surgical approach. With a 100% success rate in laparoscopic procedures, minimal post-operative complications, and a mortality rate of 1%, our findings support laparoscopic colorectal surgery as a viable and effective option for elective colorectal operations. The study underscores the successful implementation of laparoscopic techniques in a newly developed surgical setup, paving the way for improved patient outcomes in regions facing the significant healthcare challenge of CRC.
